# Short-Term Effects of Ambient Ozone, PM_2.5,_ and Meteorological Factors on COVID-19 Confirmed Cases and Deaths in Queens, New York

**DOI:** 10.3390/ijerph17114047

**Published:** 2020-06-05

**Authors:** Atin Adhikari, Jingjing Yin

**Affiliations:** Department of Biostatistics, Epidemiology & Environmental Health Sciences, Jiann-Ping Hsu College of Public Health, Georgia Southern University, Statesboro, GA 30460, USA

**Keywords:** COVID-19, SARS-CoV-2, air pollution, PM_2.5_, ozone, coronavirus, respiratory viral infections, meteorological factors, temperature, humidity

## Abstract

The outbreak of coronavirus disease 2019 (COVID-19), caused by the virus SARS-CoV-2, has been rapidly increasing in the United States. Boroughs of New York City, including Queens county, turn out to be the epicenters of this infection. According to the data provided by the New York State Department of Health, most of the cases of new COVID-19 infections in New York City have been found in the Queens county where 42,023 people have tested positive, and 3221 people have died as of 20 April 2020. Person-to-person transmission and travels were implicated in the initial spread of the outbreaks, but factors related to the late phase of rapidly spreading outbreaks in March and April are still uncertain. A few previous studies have explored the links between air pollution and COVID-19 infections, but more data is needed to understand the effects of short-term exposures of air pollutants and meteorological factors on the spread of COVID-19 infections, particularly in the U.S. disease epicenters. In this study, we have focused on ozone and PM_2.5_, two major air pollutants in New York City, which were previously found to be associated with respiratory viral infections. The aim of our regression modeling was to explore the associations among ozone, PM_2.5_, daily meteorological variables (wind speed, temperature, relative humidity, absolute humidity, cloud percentages, and precipitation levels), and COVID-19 confirmed new cases and new deaths in Queens county, New York during March and April 2020. The results from these analyses showed that daily average temperature, daily maximum eight-hour ozone concentration, average relative humidity, and cloud percentages were significantly and positively associated with new confirmed cases related to COVID-19; none of these variables showed significant associations with new deaths related to COVID-19. The findings indicate that short-term exposures to ozone and other meteorological factors can influence COVID-19 transmission and initiation of the disease, but disease aggravation and mortality depend on other factors.

## 1. Introduction

The outbreak of COVID-19 started in Wuhan, China, in December 2019 [1], and spread widely in many other countries, including Italy, Iran, Spain, UK, and the USA, during the first four months of 2020. This outbreak was declared a pandemic by the World Health Organization (WHO, Geneva, Switzerland) on 11 March 2020 [2]. As of 23 April 2020, the WHO had reported 2,631,839 confirmed cases and 182,100 confirmed deaths related to COVID-19 outbreak, from 213 countries, areas, or territories of the world [2]. On the same day, the Centers for Disease Control and Prevention (CDC) in the United States reported 865,585 confirmed cases and 48,816 confirmed deaths related to COVID-19 from 50 U.S. states, District of Columbia, Guam, the Northern Mariana Islands, Puerto Rico, and the U.S. Virgin Islands [3]. Among different COVID-19 affected states in the USA, New York state has remained at the top where 271,590 patients have tested positive and 16,162 deaths have been reported as of 23 April 2020 by the New York Department of Health [4]. We conducted this study in Queens county of New York because most cases of COVID-19 have been reported from this county (considered as the epicenter of epicenters by some news media) where 46,387 people had tested positive as of 23 April 2020 [4] and air quality of this county is poor with respect to high ozone days (received Grade F from the American Lung Association [5]).

We need a better understanding of the factors affecting the transmission of SARS-CoV-2 in order to control the rapid spread of COVID-19. Preliminary investigations on the origin of COVID-19 caused by the SARS-CoV-2 coronavirus suggests a zoonotic origin [6] because other coronavirus-related diseases, such as Middle East respiratory syndrome (MERS) and severe acute respiratory syndrome (SARS) created outbreaks due to human–animal interactions. COVID-19 early transmission-related studies in China have indicated that person-to-person transmission was a possible pathway [7,8,9], but related findings in the USA are somewhat different. Burke et al. [10] conducted a study on active monitoring of persons exposed to patients with confirmed COVID-19 in the USA. They found that person-to-person transmissions and travel-related transmissions had been documented during the early phase of the disease spread, but not for the later phase. An increasing number of newly diagnosed confirmed and presumptive COVID-19 cases were reported after February 28 for the patients without a relevant travel history or epidemiologic links to other infected patients [10].

This vital observation encouraged us to explore other alternate pathways and factors responsible for COVID-19 transmission in Queens, New York. Recently, two recent studies from China reported that short-term exposures of PM_2.5_, PM_10_, CO, NO_2_, O_3_, and ambient temperature were significantly associated with COVID-19 confirmed cases [11,12]. To our knowledge (as of 24 April 2020), one study in the USA (published preprint, not peer-reviewed yet) explored associations of long-term exposures to PM_2.5_ with mortality related to COVID-19 [13], but the way in which short-term exposures to PM_2.5_ and ozone affect COVID-19 in disease epicenters have not yet been explored in the USA. The findings of the new studies from China [11,12] and the already known poor air quality of Queens county in New York with respect to high ozone days encouraged us to explore the associations among ozone, PM_2.5_, and meteorological factors with COVID-19 confirmed cases and deaths.

Ozone is a common oxidant gas in urban air, and exposure to ozone can induce oxidative stress causing airway inflammation and increased respiratory morbidities [14,15]. A few previous studies have indicated that ozone-induced oxidative stress could alter the airway environment leading to broadened cellular tropism or susceptibility to viral infections [16,17,18]. Kesic et al. [19] showed that ozone exposure disturbed the protease/antiprotease balance in the airway liquid, and acute exposure to ozone inversely altered the expression levels of human airway proteases, which could mediate penetration of influenza viruses into host cells through cleavage of viral membrane protein hemagglutinin. In addition to these mechanistic links, a few previous epidemiological studies have shown associations among increased ambient ozone levels and respiratory viral infections [20,21,22]. 

Although the air quality in Queens is relatively better concerning PM_2.5_ (received Grade A from the American Lung Association [5]) than ozone, we wanted to explore the associaitom of PM_2.5_ with COVID-19 because the online preprint of a recent U.S. study [13] reported that an increase of only one µg/m^3^ in PM_2.5_ was associated with an 8% increase in the COVID-19 death rate (95% CI 2% and 15%). Additionally, some previous studies showed that exposure to urban airborne particulate matter altered the macrophage-mediated inflammatory response to respiratory viral infection [23] and a recent epidemiological study concluded that short-term elevated PM_2.5_ exposure was associated with higher healthcare use for acute lower respiratory infection in young children, older children, and adults [24].

We also focused on different meteorological factors in our analyses, because several previous studies have explored the associations among temperature [25,26,27], relative humidity [25,26,27], and sunlight UV-B radiations [27] and respiratory viral infections; and a few recent studies (including non-peer reviewed preprints published online) found significant associations among these factors and COVID-19 transmission and related deaths [12,28,29,30,31].

On the basis of these previous observations and knowledge gaps, we hypothesize that ambient levels of ozone, PM_2.5_, and meteorological factors in Queens county, New York could be significantly associated with COVID-19 confirmed cases and deaths. Our specific aims were to conduct univariate analyses and negative binomial regression modeling to explore the associations among ozone, PM_2.5_, daily meteorological variables (wind speed, temperature, relative humidity, and absolute humidity, cloud, and precipitations) and COVID-19 confirmed new cases and numbers of new deaths in Queens, during March and April 2020.

## 2. Materials and Methods 

### 2.1. Study Area, Air Pollutant, and Meteorological Data Collection Sites, and COVID-19 Data Collection

**Figure 1:**
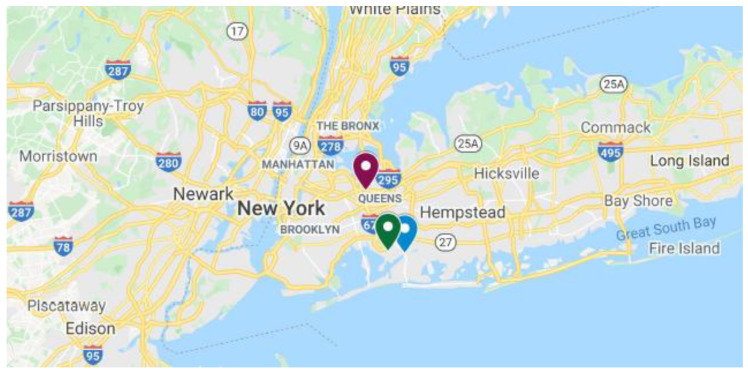
The study area and air and meteorological sampling sites in Queens county, New York. Red marker, Queens College; blue marker, Meadowmere Park; green marker, John F. Kennedy International Airport (original Google map has been personalized following Google guidelines, map copyright: Google, 2020).

The study area and sampling sites in Queens county, New York, are shown in Figure 1. Queens is a borough of New York City, which is coterminous with Queens County (but without a county government) in the state of New York, and it is the largest borough among the five boroughs of New York City [32]. Queens is adjacent to the borough of Brooklyn, at the western end of Long Island, and Nassau County is to its east (Figure 1). According to a recent U.S. census report, the borough of Queens is the second largest in population in New York state, with an estimated population of 2,253,858 residents in 2019 and approximately 47.5 percent of these residents are foreign born [32]. According to media reports, New York City continued to be the epicenter of the coronavirus pandemic in USA, in April 2020, and within the city, some of the minority neighborhoods in Queens were the most affected areas [33].

Data on daily maximum eight-hour ozone, daily average PM_2.5_, average temperature, wind speed, precipitation, and relative humidity and cloud percentages were collated from the databases of the monitoring stations at Queens College (US EPA Air Quality System, US EPA, Washington, D.C., USA), weather observation station of the NOAA National Centers for Environmental Information at John F. Kennedy International Airport, and World Weather Online, reported for the nearby Meadowmere Park area in Queens. Since PM_2.5_ data was available from two sampling locations at the same site, we used the average of the two datasets. Absolute humidity was calculated using relative humidity, temperature, and pressure data.

Data on confirmed COVID-19 cases and numbers of related deaths for Queens county were collected from USAFacts [34], which is a not-for-profit, nonpartisan civic initiative providing the most comprehensive and understandable source of government data available in the USA. According to USAFacts, the county-level data on COVID-19 is confirmed by referencing state and local agencies directly.

### 2.2. Statistical Analyses of Collected Data

The outcome of this study was that there were new COVID-19 cases and deaths every day from 1 March to 20 April 2020. In this study, the negative binomial regression model was applied for modeling the effects of two air pollutants and six meteorological factors on new cases and deaths as count outcomes with potential overdispersion (i.e., the variance of outcome is larger than the mean, which is usually the case for skewed count outcomes). Since the outcome of new deaths had many zero values (47%), we further applied the hurdle regression model to account for excess zeros. The hurdle model has two separate parts, which assumes truncated (at zero) negative binomial distribution for the non-zero counts and binomial distribution for the zeros. The predictors of interest were PM_2.5_, ozone, and some meteorological factors, i.e., wind speed, temperature, precipitation, cloud percentage, relative humidity, and absolute humidity. Note that ozone data was unavailable for two days resulting in two missing values, which was imputed by the average of the t − 1 day and the t + 1 day for the missing value at t day before fitting the models. We used 21-day moving average concentrations of PM_2.5_ and meteorological factors (i.e., the average values of the current day and previous 21 days) to represent the cumulative lag effect of these variables on disease outcomes over the past 21 days. We chose 21 days, since the incubation period of COVID-19 under conservative assumptions is about 14 days [35], and the worst-case maximum incubation period for COVID-19 can be 19 and 27 days based on available literature [36,37]. In addition, we wanted to summarize mostly the lag effect of air pollutants and meteorological factors by setting the anticipated effect time one week earlier than the 14-day incubation time for typical COVID-19 cases.

We adjusted the effect of PM_2.5_, ozone, and other meteorological factors by two confounders, i.e., lagged outcome and day trend. The lagged outcome was included to account for the potential autocorrelation of the time series of new cases (deaths), and we used the logarithm values of COVID-19 new case (or death) counts plus one, reported on the t − 1 day (i.e., log(caselag1 + 1) or log(deathlag1 + 1)). We added 1 before taking the log to avoid the case of log (0) because, at the earlier month of March 2020, there were days with zero values for both outcomes. In addition, we included a trend variable as the other confounder to control for the unobserved trend of the time series at each day (i.e., whether an increase or decrease over time). Furthermore, we checked if there was any potential seasonal pattern that existed for both time series (new cases and deaths). For example, there could be a weekly pattern due to varying testing availability and case registrations between days during the week and on weekends. However, our data did not show such a pattern, which indicated that the disease progression and management of disease testing by health departments were incessant on the weekends. To avoid the potential collinearity issue among the meteorological factors, eight single-factor (PM_2.5_, ozone, or any meteorological factor one at a time) models of the case and death outcomes, respectively, were fitted by adjusting the two confounders trend and lag outcome values, resulting in sixteen single models in total. For the death outcome, the logit/binary part of the hurdle model used the same predictors as the truncated-zero count part. Effect estimates were calculated as the exponential form of regression coefficients, which demonstrates the incidence rate ratio (IRR) of one unit increase in PM_2.5_, ozone, and meteorological variable concentrations for daily COVID-19 new cases or deaths. In addition, we interpreted the IRRs in terms of the percent (%) change of the outcomes. We performed model diagnostics based on the Durbin–Watson test of the residuals, in order to make sure that there was no significant temporal autocorrection of model residuals. We also checked whether the models fitted the observed data well, including excess zeros in the death outcome.

All analyses in this study were conducted using R statistical software (R Foundation for Statistical Computing, Vienna, Austria). A *p*-value of <0.05 was considered to be statistically significant. We used “MASS” R - package [38] to fit the negative binomial regression models, “pscl” package [39,40] to fit the hurdle regression models and “DHARMa” package [41] to perform model diagnostics on the fitted models.

## 3. Results and Discussions

### 3.1. Descriptive Analysis of Data on COVID-19 Cases and Deaths, Ozone, PM_2.5_, and Meteorological Factors

**Figure 2:**
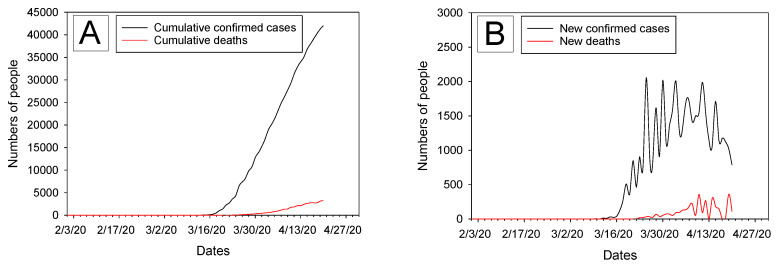
Daily variations of COVID-19 in Queens, New York between 1 February and 20 April 2020. (**A**) Cumulative confirmed cases and deaths; (**B**) New confirmed cases and deaths.

The first confirmed COVID-19 case in Queens county was reported on 7 March 2020 and as of 20 April, confirmed cumulative cases were 42,023 (mean ± SD 13,329 ± 14,671, median 7362, interquartile range (IQR) 26222). The number of daily new cases during this period ranged from 0 to 2056 (mean ± SD 824 ± 698, median 906, IQR 1395). Figure 2A,B shows trends for daily cumulative COVID-19 confirmed cases and new cases, respectively. The first deaths related to COVID-19 were reported on 22 March 2020 for 21 persons, and cumulative deaths as of 20 April 2020 were 3221 (mean ± SD 725 ± 1024, median 124, IQR 1344). The number of daily new deaths during that period ranged from 0 to 360 (mean ± SD 110 ± 104, median 71). Figure 2A,B also shows trends for daily cumulative deaths related to COVID-19 and daily new deaths, respectively.

**Figure 3:**
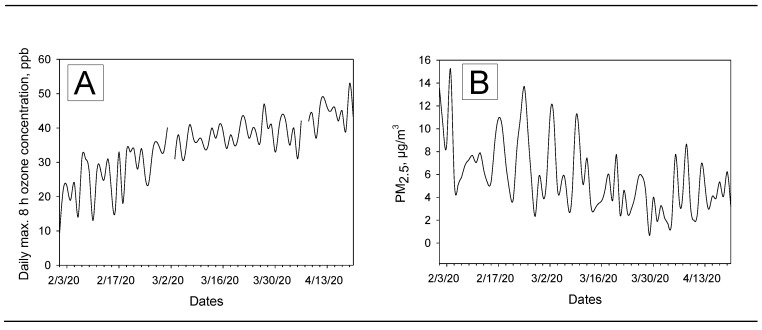
Daily variations of ozone (**A**) and PM_2.5_ (**B**) in Queens, New York between 1 February and 20 April 2020.

The ozone level (daily maximum eight-hour concentration) was found to be gradually increased during the observation period. The ozone levels ranged from 0.031 to 0.053 ppm (mean ± SD 0.04 ± 0.005 ppm, median 0.04 ppm, IQR 0.01). Daily changes of ozone levels are presented in Figure 3A. As shown in Figure 3B, a gradual decrease of PM_2.5_ levels was found probably due to no travel and stay-at-home recommendations/orders from the state government.

**Figure 4:**
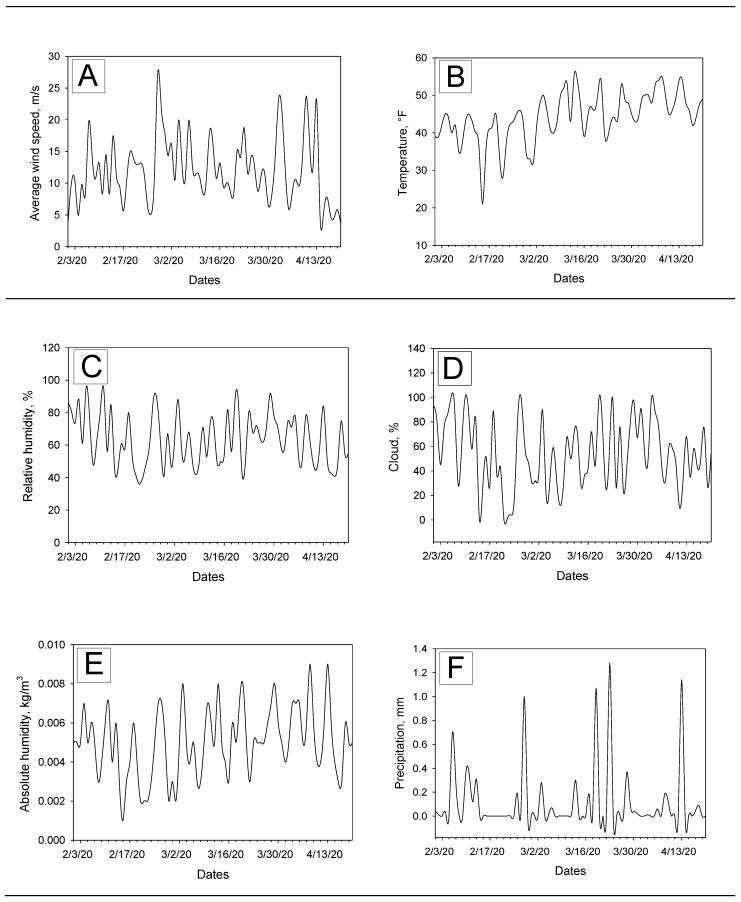
Daily variations of meteorological factors in Queens, New York between 1 February and 20 April 2020. (**A**) Wind speed; (**B**) Temperature; (**C**) Relative humidity; (**D**) Cloud; (**E**) Absolute humidity; (**F**) Precipitation.

The PM_2.5_ levels ranged from 0. 65 to 11.15 µg/m^3^ during the observation period (mean ± SD 4.73 ± 2.39 µg/m^3^, median 4.1 µg/m^3^, IQR: 2.85). Daily changes of wind speed, temperature, relative humidity, cloud percentages, absolute humidity, and precipitations are presented below in the Figure 4. Descriptive analysis results for these meteorological variables are as follows (also summarized in the Table 1): (1) Wind speed range from 3.61 to 23.71 m/s, mean ± SD 12.11 ± 5.94 m/s, median 11.41 m/s, IQR 6.27; (2) temperature range from 32 to 55 °F, mean ± SD 47.08 ± 4.97 °F, median 47.5 °F, IQR 6; (3) relative humidity range from 41% to 92%, mean ± SD 62.9% ± 13.96%, median 61%, IQR 61; (4) cloud range from 10% to 99%, mean ± SD 53.88% ± 24.18%, median 50%, IQR 39; (5) absolute humidity range from 0.002 to 0.009 kg/m^3^, mean ± SD 0.005 ± 0.001 kg/m^3^, median 0.005 kg/m^3^, IQR 0.002; and (6) precipitation levels range from 0 to 1.28 mm, mean ± SD 0.1 ± 0.28 mm, median 0 mm, IQR 0.06.

Although ozone levels were found to be increasing during the COVID-19 outbreak in Queens, none of the data points for the height-hour max ozone exceeded the EPA health-related regulatory standard of 0.07–0.085 ppm (unhealthy for sensitive groups). The ways in which lower doses of ozone are related to respiratory infections are relatively unknown, but a recent nationwide study found that older adults faced a higher risk of premature death even when the ozone level remained well below the current national standard [42]. Similarly, PM_2.5_ levels were much lower than the EPA 24-hour standard of 35 μg/m^3^ based on the three-year average of the annual 98th percentile concentrations [43]. However, we were curious to examine the effects of these low PM_2.5_ levels, because a recent non-peer reviewed preprint reported that exposure to PM_10_ could significantly enhance RNA virus infection such as N.D.V., H1N1 (PR-8), and H5N1 in human lung epithelial A459 cells [44] by increasing viral replications. Temperature levels were gradually increasing during the observation period (Figure 4B) but were mostly observed between 30 and 50 °F. This early spring temperature range was slightly higher than the last year (when March first week’s data were compared), but it was still below 70 °F at which viral lipid-dependent attachment to host cells [45] could be facilitated [46]. We certainly need more research on this issue.

### 3.2. Correlations among Ozone, PM_2.5_, and Meteorological Variables

We have examined correlations of selected pollutants and meteorological factors by calculating Spearman’s rank correlation coefficients (Table 2). We found that there were strong correlations among the predictor variables. Therefore, we could only fit single-predictor regression models adjusting the confounders in Section 3.3, whereas a multiple regression model could not provide valid results due to largely inflated standard errors of the regression estimates. 

### 3.3. Relationships among COVID-19 Confirmed Cases/Deaths, Ozone, PM_2.5_, and Meteorological Variables

The effects of ozone, PM_2.5_, and meteorological variables from 16 single-predictor models were summarized in Table 3 for new confirmed COVID-19 cases and in Table 4 for new deaths in Queens. The Durbin–Watson test *p*-values of all single-predictor models are greater than 0.05, suggesting no indication of temporal autocorrelation of model residuals.

**Figure 5:**
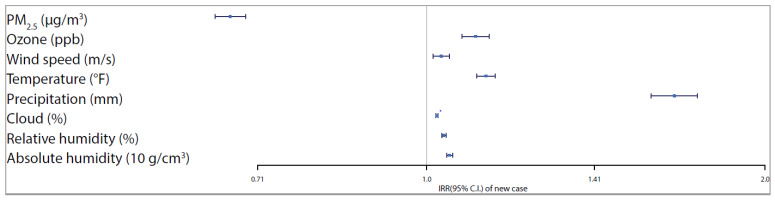
Trend and lag-case adjusted IRRs (and 95% CIs) associated with each environmental predictors for new COVID-19 confirmed cases.

**Figure 6:**
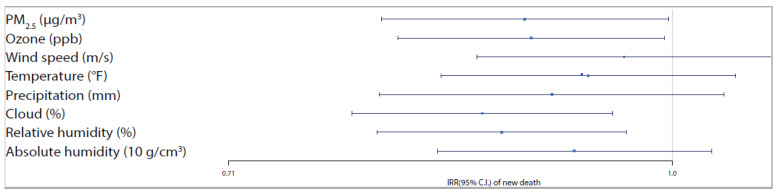
Trend and lag-case adjusted IRRs (and 95% CIs) associated with each environmental predictors for new COVID-19 related deaths.

From Table 3, we observe significant positive associations of ozone and all meteorological factors and a significant negative association among PM_2.5_ and new daily confirmed COVID-19 cases. A one-unit increase in the moving average of PM_2.5_ (µg/m^3^) was associated with a 33.11% (95% CI 31.04–35.22) decrease in the daily new COVID-19 cases. While a one-unit increase in the moving average of ozone (ppb), wind speed (m/s), temperature (°F), precipitation (mm), cloud (%), relative humidity (%), and a ten-unit increase in absolute humidity (g/cm^3^) values, during the past 21 days, was associated with a 10.51% (7.47–13.63), 3% (1.28–4.73),12.87% (10.76–15.02), 66.06% (58.33–74.17), 2.11% (1.85–2.37), 3.54% (3.09–3.99) and 4.76% (4.11–5.42) increase in the daily new COVID-19 cases. For a better comparison of effects across predictors, Figure 5 and Figure 6 show the adjusted IRRs and the corresponding 95% confidence intervals associated with each environmental predictor for new COVID-19 cases and deaths, respectively. It is clear that the results of cases are much more accurate (narrower CIs) than that of deaths due to a large number of zero values and small counts in death outcomes.

Table 3 data also suggests that every 10 g/cm^3^ increase in absolute humidity is significantly associated with 2.13% increase of new COVID-19 cases. This finding is interesting and matching with the recent preprint from the Harvard Medical School on the role of absolute humidity on transmission rates of the COVID-19 outbreak [47].

We have noticed another interesting finding in our study. Mostly, there is a significant negative trend effect showing that the count of new cases is decreasing over time. This finding is consistent across all five single-predictor models of PM_2.5_, wind speed, precipitation, and relative humidity against new cases. The trend effect in models with absolute humidity and temperature were not significant, while in the model of cloud as the predictor, it was significantly positive. This is certainly a piece of positive news when we have high levels of anxiety due to the COVID-19, and more and more countries are in lockdown, and an increasing number of people are living in isolation. However, we have to consider the limitations in the availability of test kits and limited numbers of tests conducted at the beginning of the outbreak, which could influence this finding.

Similar to our findings, a recent study from China also reported that the rise of temperature was significantly associated with an increase in daily confirmed cases of COVID-19 in Chinese cities [12]. The authors reported that a 1 °C rise in the mean temperature of last weeks (when <3 °C) was associated with a 4.861% increase in the daily COVID-19 confirmed cases. We found a more robust association (1 °F increase of temperature was associated with 10.28% increase of new cases) when temperatures of the last 21 days were considered.

A recent study in China [11] also explored short-term effects of air pollutants and meteorological factors on COVID-19 and, unlike our study, found a significant effect of PM_2.5_ on COVID-19 cases. On the one hand, this study was of a larger scale that considered data from multiple cities, which was a positive aspect. On the other hand, combining data of multiple cities involving multiple monitors operated by different regulatory agencies and local confounding factors related to air sampling and pollutant analysis methods at different locations could increase the chances of misrepresentation of data for local conditions. The model selected in the study from China [11] was interesting but needs further validation. For example, the model assumed normal distribution of data, whereas the count outcomes in COVID-19 studies were not naturally symmetrical or normally distributed although the authors tried to linearize the skewed count data by taking the logarithms. In addition, such a model cannot fit zero values since it is not valid to take logarithms on an outcome with any zero values. A more appropriate family of statistical models called generalized linear models (GLM) are available to fit the count data and the Poisson model is the most well known. In our study, we applied another member of the GLM family, the negative binomial model, because it fits overdispersed count data better than the Poisson model. We further improved the negative binomial model for the new COVID-19 deaths since approximately half of the data were zeros and regular count models generally predict a lower number of zeros for data of excess zeros. Therefore, we used the hurdle model to fit the death outcome with excess zero values. Finally, we checked the fit of the model and performed model diagnostics on the residuals to make sure that all model assumptions were valid.

Our Table 3 data also show that when cloud, precipitation, and wind data were treated with the single predictor models, all of these data were significantly associated with one-day lagged COVID-19 confirmed cases. An increase of cloud percentages and precipitations are obviously associated with decreased sunshine duration and previous epidemiological [48], and new experimental [49] findings have shown that sunlight levels are inversely correlated with influenza transmission. Therefore, our findings seem plausible.

To the best of our knowledge, this is the first study in the USA on short-term associations among two common urban air pollutants and meteorological factors with confirmed COVID-19 new cases and deaths. This study has, however, several limitations. First, the sample size is small. Secondly, the COVID-19 progress in the USA, including Queens county and New York City, has still been unpredictable for the following months. We expect a second wave of the disease and our study was concerned with only the initial disease spread period. Third, this study is limited to two major urban air pollutants, i.e., ozone and PM_2.5_. There are other gaseous pollutants, such as NO_2_ and SO_2_, which could influence transmission and pathogenesis of COVID-19. Fourth, a 21-day moving average is rather long concerning the whole study period, which was less than two months. Finally, the uncertainty of exposure levels is a problem. We used the data collected by stationary monitors. These data do not represent actual personal exposures for the people who are infected with COVID-19 and residing away from the monitoring stations.

Despite these limitations, our study findings indicate that interactions among air pollutants and meteorological factors could be responsible for the transmission and pathogenesis of COVID-19, and future large-scale studies should be designed to understand these interactions. Pollutants such as ozone could have direct effects during a critical stage of infection initiation or replication of SARS-CoV-2 viruses, which should be examined in properly designed laboratory experiments. Human susceptibility to COVID-19 could be altered by air pollutants. Pollutants could also affect the lower respiratory tract protease-antiprotease balance and microflora which could be associated with disease development for COVID-19. All these emerging research areas related to COVID-19 should be addressed soon in order to successfully manage similar viral outbreaks in the future.

## 4. Conclusions

Overall, our study findings conclude that short-term exposures of ozone and other meteorological factors in Queens county could be associated with COVID-19 transmission and initiation of the disease during the observation period till 20 April 2020, but disease aggravation and mortality depend on other factors.

## Figures and Tables

**Table 1:** Descriptive statistics of PM_2.5_, ozone, and meteorological variables across all days.

Meteorological Variables	Mean	SD	Min.	Max.
PM_2.5_ (µg/m^3^)	4.733	2.398	0.650	11.150
Ozone (ppm)	0.040	0.005	0.031	0.053
Wind speed (m/s)	12.114	5.050	3.610	23.710
Temperature (°F)	47.088	4.976	32.000	55.000
Precipitation (mm)	0.106	0.280	0.000	1.280
Cloud (%)	53.882	24.188	10.000	99.000
Relative humidity (%)	62.902	13.967	41.000	92.000
Absolute humidity (kg/m^3^)	0.005	0.002	0.002	0.009

**Table 2:** Pairwise Spearman correlation of selected pollutants and meteorological factors.

Var1	Var2	Spearman’s ρ	*p* Value
PM_2.5_	Ozone	−0.8174	<0.0001
PM_2.5_	Wind speed	0.5846	<0.0001
Ozone	Wind speed	−0.5391	<0.0001
PM_2.5_	Temperature	−0.7562	<0.0001
Ozone	Temperature	0.9661	<0.0001
Windspeed	Temperature	−0.5381	<0.0001
PM_2.5_	Precipitation	−0.5587	<0.0001
Ozone	Precipitation	0.3277	0.0177
Wind speed	Precipitation	−0.5687	<0.0001
Temperature	Precipitation	0.3530	0.0103
PM_2.5_	Cloud	−0.7975	<0.0001
Ozone	Cloud	0.8391	<0.0001
Wind speed	Cloud	−0.6048	<0.0001
Temperature	Cloud	0.7965	<0.0001
Precipitation	Cloud	0.6382	<0.0001
PM_2.5_	Relative humidity	−0.7415	<0.0001
Ozone	Relative humidity	0.7705	<0.0001
Wind speed	Relative humidity	−0.5700	<0.0001
Temperature	Relative humidity	0.7516	<0.0001
Precipitation	Relative humidity	0.6986	<0.0001
Cloud	Relative humidity	0.9727	<0.0001
PM_2.5_	Absolute humidity	−0.7911	<0.0001
Ozone	Absolute humidity	0.9478	<0.0001
Wind speed	Absolute humidity	−0.5327	<0.0001
Temperature	Absolute humidity	0.9618	<0.0001
Precipitation	Absolute humidity	0.4335	0.0013
Cloud	Absolute humidity	0.8867	<0.0001
Relative Humidity	Absolute humidity	0.8658	<0.0001

**Table 3:** Single-predictor regression model results for the relation among the moving average of lag 0–21 days of each environmental predictors and new COVID-19 cases adjusted by trend and lag 1-day values of cases.

	Estimate	Std. Error	Z Score	IRR	95% CI Lower	95% CI Upper	Pr(>|z|)
PM_2.5_ (µg/m^3^)	−0.4029	0.0160	−25.2200	0.6684	0.6478	0.6896	<0.0001
Ozone (ppb)	0.0999	0.0142	7.0179	1.1051	1.0747	1.1363	<0.0001
Wind speed (m/s)	0.0295	0.0085	3.4566	1.0299	1.0128	1.0473	0.0005
Temperature (°F)	0.1210	0.0096	12.5792	1.1287	1.1076	1.1502	<0.0001
Precipitation (mm)	0.5072	0.0243	20.8445	1.6606	1.5833	1.7417	<0.0001
Cloud (%)	0.0209	0.0013	16.0021	1.0211	1.0185	1.0237	<0.0001
Relative humidity (%)	0.0348	0.0022	15.6379	1.0354	1.0309	1.0399	<0.0001
Absolute humidity (10 g/cm^3^)	0.0465	0.0032	14.6070	1.0476	1.0411	1.0542	<0.0001

**Table 4:** Single-predictor regression model results for the relation among the moving average of lag 0–21 days of each environmental predictors and new COVID-19 deaths adjusted by trend and lag 1-day values of deaths.

	Estimate	Std. Error	Z Score	IRR	95% CI Lower	95% CI Upper	Pr(>|z|)
PM_2.5_ (µg/m^3^)	−0.1151	0.0573	−2.0106	0.8912	0.7966	0.9971	0.0444
Ozone (ppb)	−0.1101	0.0531	−2.0722	0.8958	0.8072	0.9941	0.0382
Wind speed (m/s)	−0.0375	0.0587	−0.6396	0.9632	0.8585	1.0806	0.5224
Temperature (°F)	−0.0655	0.0587	−1.1160	0.9366	0.8349	1.0508	0.2644
Precipitation (mm)	−0.0941	0.0688	−1.3687	0.9102	0.7954	1.0415	0.1711
Cloud (%)	−0.1484	0.0521	−2.8469	0.8621	0.7784	0.9548	0.0044
Relative humidity (%)	−0.1334	0.0497	−2.6838	0.8752	0.7939	0.9647	0.0073
Absolute humidity (10 g/cm^3^)	−0.0764	0.0547	−1.3962	0.9264	0.8322	1.0313	0.1626
